# Alcohol Intake, Cardiometabolic Risk, Fibrosis, and Gut Microbiota in Steatotic Liver Disease: A Population-Based Health Checkup Study

**DOI:** 10.3390/jcm15082860

**Published:** 2026-04-09

**Authors:** Keisuke Furusawa, Chikara Iino, Keita Mikami, Satoshi Sato, Kenta Yoshida, Shigeyuki Nakaji, Tatsuya Mikami, Hirotake Sakuraba

**Affiliations:** 1 Department of Gastroenterology, Hematology, and Clinical Immunology, Hirosaki University Graduate School of Medicine, Hirosaki 036-8562, Japan; fk-9562@hirosaki-u.ac.jp (K.F.); km_1522@hirosaki-u.ac.jp (K.M.); satoshis@hirosaki-u.ac.jp (S.S.); kyoshida@hirosaki-u.ac.jp (K.Y.); hirotake@hirosaki-u.ac.jp (H.S.); 2 Department of Preemptive Medicine, Innovation Center for Health Promotion, Hirosaki University Graduate School of Medicine, Hirosaki 036-8562, Japan; nakaji@hirosaki-u.ac.jp (S.N.); tmika@hirosaki-u.ac.jp (T.M.)

**Keywords:** steatotic liver disease, MASLD, MetALD, alcohol intake, cardiovascular risk, transient elastography, liver fibrosis, gut microbiota

## Abstract

**Background**: The real-world risk profiles of newly defined steatotic liver disease (SLD) subtypes—MASLD, MetALD, and ALD—remain incompletely described in community settings. **Methods**: A cross-sectional analysis of 950 health-checkup participants was conducted. SLD (CAP ≥ 248 dB/m) and significant fibrosis (LSM ≥ 7.0 kPa) were evaluated by transient elastography. Associations between alcohol intake, cardiometabolic factors, fibrosis, and gut microbiota (16S rRNA sequencing) were assessed. **Results**: Among 950 participants, 310 (33%) had SLD (MASLD, *n* = 222; MetALD, *n* = 41; ALD, *n* = 23). Treated as a continuous exposure, higher alcohol intake was significantly correlated with elevated systolic/diastolic blood pressure, triglycerides, AST, and γ-GTP, but inversely correlated with HOMA-IR (all *p* < 0.05). In multivariable logistic regression adjusting for cardiometabolic factors, BMI was the only independent predictor of fibrosis (adjusted OR 1.22, 95% CI 1.11–1.35, *p* < 0.01), whereas alcohol intake showed no independent association. Furthermore, microbiota analysis revealed that ALD-related SLD was characterized by significant depletion of *Blautia* and enrichment of *Gemella* (FDR q < 0.05) compared to non-SLD controls, indicating an alcohol-associated dysbiosis signature. **Conclusions**: In early-stage SLD, alcohol intake continuously exacerbates cardiometabolic risk factors, whereas fibrosis is predominantly driven by BMI. These findings support quantitative alcohol/BMI integration for risk stratification, alongside microbiota profiling to detect ALD-related dysbiosis.

## 1. Introduction

Steatotic liver disease (SLD) has recently been redefined as an umbrella term that encompasses a spectrum of fatty liver conditions, with metabolic dysfunction-associated steatotic liver disease (MASLD) replacing the former term non-alcoholic fatty liver disease (NAFLD). A global consensus process in 2023 proposed MASLD as the central category for steatosis in the presence of cardiometabolic risk factors, and introduced MetALD and alcohol-related liver disease (ALD) for phenotypes in which higher alcohol intake coexists with metabolic dysfunction [1,2,3,4]. In this framework, MASLD largely overlaps with the historical NAFLD population and benefits from a substantial body of evidence on hepatic and extrahepatic outcomes [5,6,7,8]. By contrast, the epidemiological features, prognostic implications, and optimal risk stratification of MetALD and ALD have not been fully clarified, especially outside tertiary care settings [3,4,9,10].

NAFLD/MASLD is now recognized as an independent risk factor for atherosclerotic cardiovascular disease (ASCVD), with multiple cohort studies and meta-analyses demonstrating increased risks of cardiovascular events and cardiovascular mortality [5,6,7,8]. Recent large-scale analyses applying the new SLD subcategories have suggested that, compared with individuals without SLD, cardiovascular disease (CVD) risk increases with MASLD and may further increase in MetALD, while MetALD and ALD appear to carry higher risks for liver-related events and all-cause mortality than MASLD [4,10,11]. However, findings are not entirely consistent: some studies report similar rates of major adverse cardiovascular events (MACE) between MASLD and ALD/MetALD [4], whereas others report a graded increase in incident CVD risk across MASLD, MetALD, and ALD when competing risks are considered [11]. Thus, the degree to which CVD risk differs between SLD subtypes remains debated.

Liver fibrosis is the strongest histological predictor of liver-related and overall mortality in MASLD and related conditions [12,13]. Several recent studies have examined the prevalence and determinants of advanced fibrosis across MASLD, MetALD, and ALD, using non-invasive fibrosis markers or elastography [3,9,10,14]. Nevertheless, it is still unclear how much alcohol dose *per se*, as opposed to cardiometabolic factors such as obesity, dysglycemia, hypertension, and dyslipidemia, contributes to fibrosis risk in real-world populations. Moreover, the alcohol cut-offs currently used to define MASLD/MetALD/ALD were derived by expert Delphi consensus rather than by dose–response modeling based on observational data [1,2,3,4]. It is therefore uncertain whether these thresholds truly correspond to biologically meaningful inflection points for cardiovascular or fibrotic risk.

In parallel, growing evidence indicates that alterations of the gut microbiota and microbial metabolites are involved in both MASLD and ALD through the gut–liver axis [15,16,17,18]. Dysbiosis, including depletion of short-chain fatty acid (SCFA)-producing taxa and expansion of oral-like or potentially pro-inflammatory taxa, has been linked to intestinal barrier dysfunction, systemic inflammation, and progression of fatty liver disease [16,17,18,19,20]. However, few studies have evaluated gut microbiota patterns in SLD stratified by alcohol intake (MASLD vs. MetALD vs. ALD) in the general population.

Against this background, this study aimed to characterize how alcohol intake relates to cardiometabolic profiles, liver fibrosis, and gut microbiota among individuals with fatty liver identified in a community-based health checkup program. Specifically, alcohol intake was treated both as a categorical variable defined by MASLD/MetALD/ALD and as a continuous exposure in 10 g/day increments. Subsequently, the following were examined: (i) the distribution of cardiometabolic criteria across alcohol strata, (ii) independent determinants of fibrosis including BMI and other cardiometabolic factors, and (iii) alcohol dose–related differences in gut microbiota. By doing so, this study sought to explore whether the current consensus cut-offs for MetALD and ALD adequately capture cardiometabolic and fibrotic risks in the setting of metabolic dysfunction-associated SLD, and to elucidate potential microbiota signatures that characterize alcohol-related SLD. The 2018 Iwaki health-checkup cohort has been previously reported in a study focused on metabolic dysfunction-associated fatty liver disease/NAFLD criteria, diet, and microbiota profiling [21]. In the present work, a distinct question is addressed by applying the 2023 SLD nomenclature (MASLD/MetALD/ALD) and alcohol-focused analyses, including multivariable microbiome modeling.

## 2. Materials and Methods

### 2.1. Study Population

The Iwaki Health Promotion Project is an ongoing community-based health checkup program conducted since 2005 in the Iwaki area of Hirosaki City, Aomori Prefecture, Japan. Each year, more than 1000 residents are enrolled through public recruitment and voluntarily participate in a comprehensive health examination. For each participant, over 800 variables are collected, including demographic characteristics, medical history, lifestyle factors, blood biochemistry, and liver-related parameters.

Participants were drawn from the 2018 Iwaki Health Promotion Project, which has been described previously. While the cohort framework and core measurements are shared with the prior publication, the present manuscript uses different disease definitions and analytic objectives centered on alcohol exposure, cardiometabolic risk, fibrosis by LSM thresholds, and multivariable microbiome associations using MaAsLin2 (Bioconductor package; Harvard T.H. Chan School of Public Health, Boston, MA, USA).

**Figure 1:**
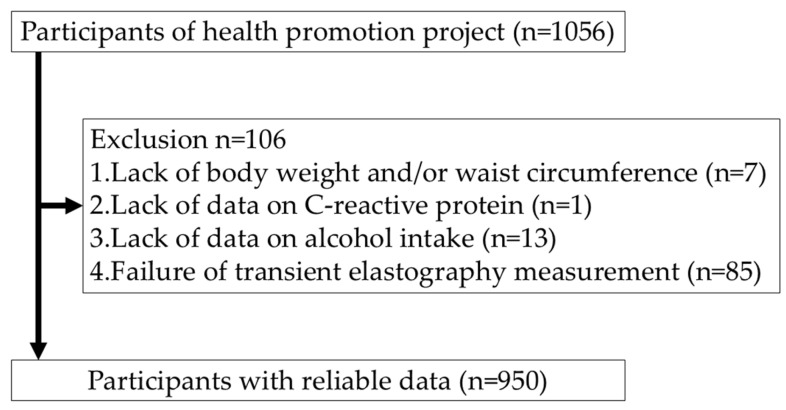
Flow diagram of participant enrollment.

In the 2018 survey, 1056 adults participated. Individuals with missing data for body weight or waist circumference (*n* = 7), missing blood tests (*n* = 1), missing alcohol intake data (*n* = 13), or unsuccessful FibroScan^®^ measurements (*n* = 85). After these exclusions, 950 participants were included in the final analysis (Figure 1).

### 2.2. Clinical and Laboratory Parameters

Recorded variables included sex, age, height, body weight, body mass index (BMI; kg/m^2^), waist circumference, systolic blood pressure (SBP), diastolic blood pressure (DBP), total daily caloric intake, alcohol consumption, and sleep duration. Blood sampling included aspartate aminotransferase (AST), alanine aminotransferase (ALT), γ-glutamyl transpeptidase (γ-GTP), fasting plasma glucose, fasting insulin, hemoglobin A1c (HbA1c), homeostatic model assessment for insulin resistance (HOMA-IR), high-density lipoprotein cholesterol (HDL-C), triglycerides (TG), platelet count, and Mac-2 binding protein glycosylation isomer (M2BPGi). Total caloric intake and alcohol consumption were estimated using the brief-type self-administered diet history questionnaire (BDHQ) [22].

### 2.3. Assessment of Hepatic Steatosis and Fibrosis

Liver stiffness (LSM) and hepatic fat content, determined via the controlled attenuation parameter (CAP), were measured by transient elastography (FibroScan^®^, Echosens, Paris, France) performed by five hepatology specialists. Examinations were considered unreliable if fewer than 10 valid measurements were obtained, or if the interquartile range (IQR) of LSM divided by the median exceeded 0.30, in line with previous recommendations [23].

Fatty liver was defined as CAP ≥ 248 dB/m [24]. Significant fibrosis was defined as LSM ≥ 7.0 kPa, a threshold commonly used to indicate at least F2 fibrosis in MASLD cohorts [24].

### 2.4. Classification of Steatotic Liver Disease Categories

Among participants with fatty liver (CAP ≥ 248 dB/m), MASLD was defined according to the recent consensus criteria as steatosis in the presence of at least one of the following cardiometabolic risk factors [1,2,3,4]:

- BMI ≥ 23 kg/m^2^ or waist circumference > 94 cm in men and > 80 cm in women;
- Fasting plasma glucose ≥ 100 mg/dL, 2-h post-load glucose ≥ 140 mg/dL, HbA1c ≥ 5.7%, or known/type 2 diabetes under treatment;
- Blood pressure ≥ 130/85 mmHg or antihypertensive treatment;
- Triglycerides ≥ 150 mg/dL or lipid-lowering therapy;
- HDL-C ≤ 40 mg/dL in men or ≤ 50 mg/dL in women, or lipid-lowering therapy.

Among MASLD cases, MetALD was defined as the presence of steatosis with metabolic dysfunction and alcohol consumption of 30–60 g/day in men or 20–50 g/day in women. ALD was defined as steatosis with excessive alcohol intake of ≥60 g/day in men or ≥50 g/day in women. Cases with hepatitis B surface antigen or hepatitis C virus antibody positivity, or with use of established steatogenic drugs (e.g., amiodarone, methotrexate, corticosteroids, tamoxifen), were classified as specific-etiology SLD. Steatotic cases not fulfilling any of the above criteria were categorized as cryptogenic SLD.

Cardiometabolic criteria and fibrosis were compared across MASLD, MetALD, and ALD, and correlations between alcohol intake and clinical factors were evaluated. In addition, these associations were analyzed by stratifying alcohol intake in 10 g/day increments to examine dose–response patterns independent of categorical cut-offs.

### 2.5. Gut Microbiota Profiling

Stool specimens were obtained during the health checkup using Metabolokeeper^®^ (TechnoSuruga Laboratory Co., Ltd., Shizuoka, Japan) [25]. Samples were then mixed with the supplied buffer, homogenized, and kept at 4 °C until DNA extraction. The gut microbial community was profiled by 16S rRNA gene amplicon sequencing targeting the V3–V4 region. In brief, genomic DNA was extracted from the stool suspensions, and the V3–V4 region was PCR-amplified with broad-range primers that enable concurrent detection of Bacteria and Archaea, as described and validated by Takahashi et al. [26]. Libraries were sequenced in paired-end mode on an Illumina MiSeq system (Illumina, San Diego, CA, USA). Resulting reads were processed to generate taxonomic assignments, and genus-level relative abundances were calculated for downstream analyses.

To evaluate differences in gut microbiota across fatty liver subtypes, genus-level profiles were first compared between participants without steatotic liver disease (not SLD) and each fatty liver subtype (MASLD, MetALD, and ALD). Alcohol-related microbial shifts within fatty liver were further assessed using subtype contrasts and alcohol intake strata. Multivariable association analyses were performed using MaAsLin2 to identify genera independently associated with SLD subtypes and alcohol exposure while adjusting for prespecified covariates. Multiple testing was controlled using the false discovery rate (FDR), and associations with an FDR-adjusted q-value < 0.05 were considered statistically significant. Genera showing significant differences between not SLD and fatty liver subtypes were prioritized for interpretation.

### 2.6. Statistical Analysis

All statistical analyses were performed using EZR (version 1.68; Saitama Medical Center, Jichi Medical University, Saitama, Japan), a graphical user interface for R [27]. Categorical variables were compared using the chi-squared test. Continuous variables across three or more groups were compared using the Kruskal–Wallis test. Pearson’s correlation coefficients were used to evaluate associations between alcohol intake and clinical variables. A two-sided *p*-value < 0.05 was considered statistically significant.

For fibrosis, multivariable logistic regression models were constructed with fibrosis (LSM ≥ 7.0 kPa) as the dependent variable, and BMI, alcohol intake, and cardiometabolic criteria (e.g., glycemic status, blood pressure, lipid parameters) as independent variables. Collinearity between covariates was assessed, and model selection was guided by clinical plausibility and parsimony. To explore very early structural changes, the multivariable logistic regression analysis was repeated using an alternative fibrosis definition of LSM ≥ 6.0 kPa. Multicollinearity among covariates was assessed using the Variance Inflation Factor (VIF), with a VIF value of < 5 considered indicative of no severe collinearity. Furthermore, a subgroup analysis stratified by BMI (<25 kg/m^2^ and ≥25 kg/m^2^) was conducted to evaluate the odds ratios for fibrosis in non-obese and obese participants.

### 2.7. Ethics

This study was conducted in accordance with the ethical standards of the Declaration of Helsinki and was approved by the Hirosaki University Medical Ethics Committee (approval no. 2025-068). All participants provided written informed consent after receiving detailed explanations of the study purpose and examination procedures.

## 3. Results

### 3.1. Baseline Characteristics and SLD Categories

Among 950 participants (402 men, 42.3%; mean age 52 ± 15 years), fatty liver defined by CAP ≥ 248 dB/m was present in 310 individuals (33%). The distribution of SLD categories was as follows: MASLD *n* = 222, MetALD *n* = 41, ALD *n* = 23, specific-etiology SLD *n* = 11, and cryptogenic SLD *n* = 13.

Mean alcohol intake was 3.0 ± 6.4 g/day in MASLD, 41.2 ± 9.9 g/day in MetALD, 74.3 ± 17.3 g/day in ALD, 9.7 ± 17.0 g/day in specific-etiology SLD, and 6.0 ± 7.7 g/day in cryptogenic SLD. Clinical characteristics of each group are summarized in Table 1.

### 3.2. Alcohol Intake and Cardiometabolic Profile

When alcohol consumption was categorized in 10 g/day increments across the entire cohort (Table 2), SBP, DBP, TG, body weight, total caloric intake, AST, and γ-GTP all showed positive associations with higher alcohol intake, suggesting a continuous worsening of cardiometabolic and hepatic biochemical profiles from low to higher drinking levels (Table 3). In contrast, HOMA-IR decreased with increasing alcohol consumption, indicating an inverse relationship between alcohol intake and insulin resistance in this relatively healthy population.

Similar trends were observed when comparisons were made among MASLD, MetALD, and ALD categories: MetALD and ALD tended to exhibit higher blood pressure, TG, and liver enzymes compared with MASLD, consistent with a more adverse cardiometabolic profile in alcohol-exposed fatty liver.

### 3.3. Fibrosis and Associated Factors

Significant fibrosis (LSM ≥ 7.0 kPa) was present in 40 participants (12.9%) among those with fatty liver. In multivariable analysis including alcohol intake and cardiometabolic criteria, BMI emerged as the only independent predictor of fibrosis, whereas alcohol consumption did not show an independent association with LSM-defined fibrosis after adjustment (Table 4). In the multivariable logistic regression full model, exact collinearity checks revealed no multicollinearity among the variables, as all VIF values were ≤ 1.31 (HOMA-IR: 1.13, systolic BP: 1.08, HDL-cholesterol: 1.16, BMI: 1.31). Furthermore, when stratified by BMI to provide additional context, the odds ratio of HOMA-R for fibrosis was 1.49 (95% CI: 0.69–3.24) in the non-obese group (BMI < 25) and 1.22 (95% CI: 0.99–1.51) in the obese group (BMI ≥ 25). In a sensitivity analysis using a lower fibrosis threshold (LSM ≥ 6.0 kPa), BMI remained independently associated with fibrosis (adjusted OR 1.21; 95% CI 1.11–1.32; *p* < 0.01), whereas HOMA-IR was attenuated and no longer statistically significant (adjusted OR 1.20; 95% CI 0.97–1.49; *p* = 0.10) (Table S1). This finding suggests that, in this community-based cohort with predominantly mild disease, fibrosis burden is more strongly anchored to obesity than to alcohol dose.

### 3.4. Gut Microbiota According to Alcohol Exposure

**Figure 2:**
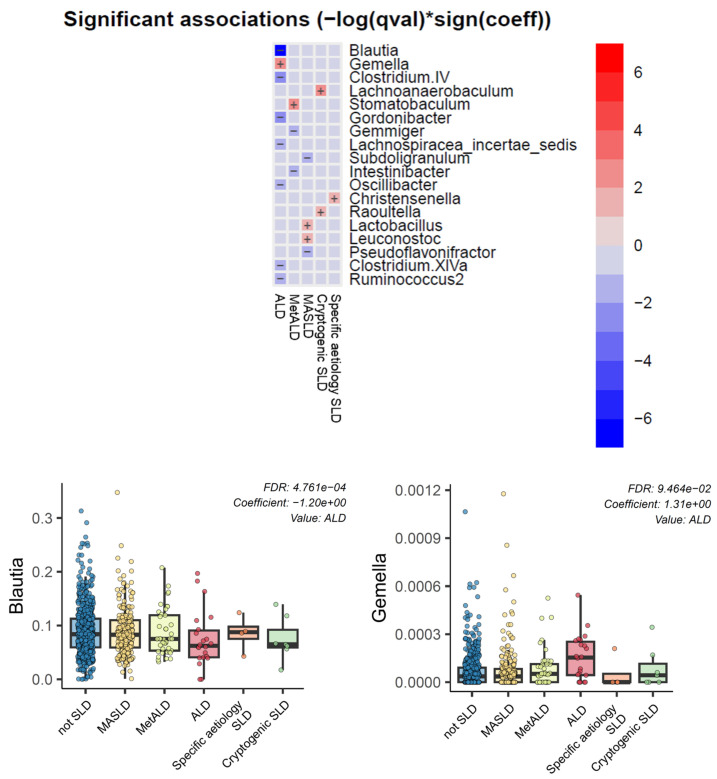
Differences in gut microbiota across fatty liver subtypes compared with participants without SLD. Significant taxa, including Blautia and Gemella, were defined as FDR q < 0.05. The *x*-axis “signed log q-value” indicates both the direction and significance of the association (positive values = enrichment; negative values = depletion). Heatmap summarizing genus-level associations identified by MaAsLin2 across SLD subtypes (MASLD, MetALD, ALD, specific-etiology SLD, and cryptogenic SLD), using the non-SLD group as the reference. Each cell displays the signed and FDR-weighted association metric (−log(q value) × sign(β)), where β denotes the model coefficient; positive values indicate higher relative abundance and negative values indicate lower relative abundance compared with the non-SLD group. Boxplots showing genus-level relative abundance across groups for *Blautia* and *Gemella*, highlighting ALD-related shifts. FDR-adjusted q values and model coefficients are shown within each panel. Boxplots indicate the median and interquartile range (IQR); whiskers represent 1.5 × IQR, and dots represent individual participants.

Gut microbiota analysis at the genus level showed significant differences according to alcohol exposure in individuals with fatty liver. Specifically, ALD-related fatty liver was characterized by a significant decrease in the relative abundance of *Blautia* and a significant increase in *Gemella* (Figure 2).

## 4. Discussion

In this community-based health checkup cohort, alcohol intake was evaluated both as a categorical determinant of MASLD/MetALD/ALD and as a continuous exposure in relation to cardiometabolic risk, fibrosis, and gut microbiota. Three main findings emerged. First, alcohol intake was positively associated with SBP, DBP, and TG from relatively low doses, whereas HOMA-IR showed an inverse association. Second, fibrosis was independently associated with BMI but not alcohol dose. Third, ALD-related fatty liver exhibited a characteristic microbiota pattern with reduced *Blautia* and increased *Gemella*, consistent with an inflammatory shift along the gut–liver axis.

### 4.1. Alcohol Intake and Cardiovascular Risk Stratification in SLD

The present data show that among individuals with fatty liver, higher alcohol intake is associated with progressively worse cardiometabolic profiles—particularly elevated blood pressure and triglycerides—forming a distinct higher-risk stratum compared with non-drinkers or light drinkers. NAFLD/MASLD has been consistently linked to increased ASCVD risk in observational studies and meta-analyses [5,6,7,8], and recent analyses using the new SLD categories suggest that cardiovascular risk increases from MASLD to MetALD [3,4,9,10]. At the same time, studies directly comparing NAFLD and alcoholic fatty liver have reported that cardiovascular hospitalization risk may be more prominent in NAFLD, whereas ALD is dominated by liver-related events and competing risks such as external causes of death [11].

Because MetALD and ALD accounted for relatively small proportions of this community cohort, analyses treating alcohol intake as a continuous exposure were prioritized to reduce reliance on small subgroup contrasts. The 10 g/day-increment analysis provides complementary evidence from a general population setting, indicating that alcohol behaves as a continuous exposure that elevates blood pressure and TG without a clear “safe threshold” around the current MASLD/MetALD or MetALD/ALD cut-offs. This is in line with large meta-analyses demonstrating near-linear increases in blood pressure and incident hypertension risk with each 10 g/day increment in alcohol consumption, without an obvious no-risk zone [28,29]. From a practical standpoint, these findings support taking a quantitative drinking history and applying early cardiovascular risk management (particularly for SBP, DBP, and TG) even in individuals whose alcohol intake remains within the nominally “allowed” range for MASLD or MetALD.

These observations do not contradict prior reports that ALD is more strongly associated with liver-related events than with cardiovascular events [3,4,11]. Rather, they complement those data by showing, in a relatively healthy SLD cohort, that alcohol intake worsens the cardiometabolic risk profile from early exposure levels. Thus, the apparent predominance of liver events in ALD might partly reflect competing risks rather than the absence of alcohol-related cardiovascular harm.

### 4.2. Alcohol Intake and Insulin Resistance

A paradoxical pattern was also observed in which alcohol intake was positively associated with blood pressure and TG but inversely associated with HOMA-IR. This counterintuitive relationship has been reported in prior studies of Japanese men, where habitual alcohol consumption was associated with lower HOMA-IR independent of obesity [30], and intervention trials have shown reductions in fasting insulin and HbA1c with moderate alcohol intake in non-diabetic individuals [31]. Our findings are therefore consistent with existing evidence that light-to-moderate drinking can lower insulin resistance indices.

However, prior work indicates that lower HOMA-IR in drinkers does not necessarily imply a favorable metabolic state. In non-obese, non-diabetic Japanese men, moderate drinkers showed lower HOMA-IR but also reduced β-cell function (HOMA-B) and higher fasting glucose than non-drinkers, suggesting that even moderate alcohol intake may accelerate β-cell dysfunction and fasting hyperglycemia [32]. Furthermore, a recent systematic review and narrative synthesis in NAFLD/MASLD patients found that any level of alcohol intake, including within recommended limits, was associated with worse liver-related outcomes [33]. Taken together, these results imply that the inverse association between alcohol and HOMA-IR should not be interpreted as evidence that drinking is metabolically protective in MASLD or SLD. In clinical practice, alcohol restriction, along with blood pressure and lipid control, remains reasonable for risk reduction even when insulin resistance markers appear “improved”.

### 4.3. Determinants of Fibrosis: BMI as a Key Anchor in Community SLD

In the multivariable models, BMI was the only cardiometabolic factor independently associated with LSM-defined fibrosis, while alcohol intake was not. This aligns with population-based data in which BMI, diabetes, and related metabolic traits are major predictors of advanced fibrosis in NAFLD/MASLD [12,14]. For example, a general population cohort from Argentina identified BMI, diabetes/hyperglycemia, and hypertriglyceridemia as independent determinants of F2 or greater fibrosis in NAFLD [12]. Analyses using NHANES data and the MASLD/MetALD/ALD framework similarly indicate that BMI and diabetes are key predictors of fibrosis severity across the SLD spectrum [14].

Conversely, some biopsy-based MASLD cohorts have found that diabetes and hypertension, rather than BMI, are independently associated with advanced fibrosis [34,35], while other studies emphasize fasting glucose or diabetes as the strongest fibrosis-related factor among multiple cardiometabolic risks [35,36]. These discrepancies likely reflect differences in disease spectrum (biopsy-proven vs. health checkup cohorts), obesity distribution, and model specification. In this community-based sample with relatively few cases of MASH or advanced fibrosis, it is plausible that overall adiposity (BMI) plays the dominant role, with other cardiometabolic factors becoming more influential in more advanced or biopsy-selected populations. When using a lower LSM threshold (≥6.0 kPa) to capture earlier structural changes, the independent association of BMI persisted, whereas the association of HOMA-IR was attenuated. This suggests that obesity is a robust anchor for fibrosis risk in this health-check setting, while metabolic indices may show threshold-dependent effects, particularly in the lower LSM range where measurement variability may be greater.

Clinically, these results suggest that in early-to-moderate SLD detected in health checkups, fibrosis risk stratification may be more effectively anchored on BMI than on alcohol dose per se. Alcohol reduction remains important, particularly for cardiovascular and hepatic outcomes, but in the present setting it did not independently stratify fibrosis beyond BMI and other factors.

### 4.4. Gut Microbiota: Blautia Depletion and Gemella Enrichment as an ALD-Related Dysbiosis Signature

A novel aspect of this study is the identification of a characteristic microbiota pattern in ALD-related fatty liver, namely decreased *Blautia* and increased *Gemella* at the genus level. *Blautia*, a member of the Firmicutes phylum, is an SCFA-producing genus with important roles in bile acid metabolism and anti-inflammatory signaling [16,17,18]. Depletion of *Blautia* and other beneficial SCFA producers has been reported in decompensated alcoholic cirrhosis, along with reduced fecal SCFA levels and a pro-inflammatory gut environment [16]. Similar depletion has been observed in NAFLD/NASH, where reduced SCFA-producing Firmicutes are linked to steatosis, inflammation, and fibrosis progression [18].

*Gemella* is an oral and proximal small-intestinal commensal that has been implicated in “oralization” of the gut microbiota in liver disease. In patients with cirrhosis, duodenal mucosal microbiota studies have shown increased abundance of *Gemella*-related operational taxonomic units, and liver tissue microbiome analyses in NAFLD have detected *Gemella* DNA in association with portal inflammation [17,19]. Moreover, multi-omics studies have reported expansion of *Gemella* and related oral taxa in cirrhosis and hepatocellular carcinoma, suggesting a link between barrier dysfunction, bacterial translocation, and disease progression [20].

The finding that ALD-related fatty liver is characterized by a reduced relative abundance of *Blautia* and an increased relative abundance of *Gemella* suggests a dysbiosis pattern that combines depletion of beneficial SCFA-producing bacteria with enrichment of oral-associated and potentially pathogenic genera. This is biologically consistent with SCFA depletion, impaired mucosal barrier function, and oral–gut–liver axis disruption in alcohol-exposed SLD. While prior studies have documented these taxa in cirrhosis and NASH [16,17,18,19,20], these results propose this combination as a potential signature already present at the fatty liver stage in ALD, before overt decompensation. Future longitudinal studies are warranted to determine whether the combination of *Blautia* depletion and *Gemella* enrichment can serve as a microbiome-based biomarker for fibrosis progression or as a target for interventions (e.g., SCFA restoration, modulation of oral–gut translocation) in alcohol-associated SLD.

### 4.5. Alcohol Cut-Offs for MetALD: Continuous Exposure Versus Categorical Thresholds

Current alcohol cut-offs for MASLD, MetALD, and ALD are based on expert consensus rather than empirical dose–response analyses [1,2,3,4]. By evaluating alcohol intake in 10 g/day increments, this study provides evidence that, at least from a cardiovascular risk factor perspective, these categorical thresholds do not coincide with sharp changes in SBP, DBP, or TG. Instead, a largely continuous deterioration in these parameters was observed across alcohol strata, with few indications of a “safe zone” around the MASLD/MetALD or MetALD/ALD boundaries. These findings echo large meta-analyses showing linear associations between alcohol dose and blood pressure or hypertension risk [28,29].

These results therefore suggest that, in community-dwelling individuals with SLD, alcohol intake should be considered as a continuous exposure when assessing cardiometabolic risk, rather than relying solely on categorical thresholds. For clinical practice and public health, this implies that even alcohol intake within the nominal “MetALD range” may already be associated with an unfavorable cardiometabolic profile. For future refinements of the SLD framework, dose–response analyses based on real-world data—including both liver- and cardiovascular-related outcomes—will be important to identify biologically meaningful thresholds, if any, rather than relying purely on consensus.

### 4.6. Limitations

This study has several limitations. First, because this study was based on a voluntary health checkup cohort, it is inherently susceptible to selection bias. Specifically, health-conscious individuals and “healthy drinkers” may be overrepresented, whereas unhealthy heavy drinkers might be less likely to participate. Consequently, the prevalence of MASH and advanced fibrosis was relatively low, and these findings may not be fully generalizable to the broader general population or hospital-based populations enriched with advanced ALD or cirrhosis. Moreover, the smaller sample sizes in the MetALD and ALD groups may have limited power for subgroup analyses, especially for detecting modest microbiome differences after multiple-testing correction. Second, the cross-sectional design precludes inference about causal relationships. Theoretically, the lack of a strong association between alcohol and advanced fibrosis, or the inverse correlation with HOMA-IR, could be influenced by reverse causality, such as the “sick quitter” effect (i.e., individuals stopping alcohol consumption due to existing health issues). However, because this cohort consists of general health checkup participants, the prevalence of individuals with severe underlying diseases who were forced to abstain from alcohol is likely very low. Thus, the actual impact of this effect on the present results is expected to be minimal, although longitudinal studies are still necessary to fully establish causality. Third, alcohol intake and dietary variables were assessed by self-reported questionnaires (BDHQ). Although validated, this method is prone to underreporting—particularly among heavy drinkers due to social desirability biases—which may have led to misclassification among the SLD subgroups. Fourth, the gut microbiota analysis was based on 16S rRNA sequencing at the genus level; species-level resolution and functional profiling by shotgun metagenomics would provide more detailed insights into microbial pathways. Finally, the study was performed in a single Japanese region, and generalizability to other ethnicities and dietary patterns is uncertain. Although the cohort overlaps with a prior publication, the research questions, definitions, and statistical analyses differ, and no tables or figures are reproduced verbatim.

## 5. Conclusions

In a Japanese general health checkup cohort with SLD, it was found that alcohol intake—treated as a continuous exposure—was associated with a dose-dependent worsening of cardiovascular risk factors (SBP, DBP, TG) from relatively low drinking levels, while fibrosis risk was more strongly linked to BMI than to alcohol dose. ALD-related SLD exhibited a characteristic dysbiosis pattern with decreased *Blautia* and increased *Gemella*, consistent with SCFA depletion and disruption of the oral–gut–liver axis.

For risk stratification, these findings support quantitative alcohol/BMI integration rather than reliance solely on consensus-based MASLD/MetALD/ALD alcohol thresholds. In the context of MASLD and concomitant illnesses, integrating cardiometabolic risk assessment with non-invasive fibrosis staging and microbiota profiling may improve early identification and management of high-risk individuals.

## Figures and Tables

**Table 1:** Characteristics of all participants.

	MASLD (*n* = 222)		MetALD (*n* = 41)		ALD (*n* = 23)		Specific Etiology SLD (*n* = 11)		Cryptogenic SLD (*n* = 13)		Not SLD (*n* = 640)	
Demographics												
Age (years)	58	(21–82)	56	(31–78)	51	(29–76)	60	(38–80)	37	(24–71)	51	(19–88)
Male, *n* (%)	91	(41.0%)	34	(82.9%)	22	(95.7%)	5	(45.5%)	6	(46.2%)	241	(37.7%)
Lifestyle factors												
Alcohol intake (g/day)	0	(0–25.5)	39.2	(20.2–59.1)	70.3	(52.3–133.2)	1.1	(0–52.7)	2.9	(0–25.9)	1.2	(0–167.9)
Caloric intake (kcal/day)	1774	(626–3402)	2022	(1143–3480)	2369	(1732–3460)	2248	(718–3097)	1766	(999–2128)	1746	(528–4001)
Anthropometric measures												
Body Weight (kg)	64.9	(38.4–133.7)	69.0	(49.4–103.8)	71.1	(60.0–99.8)	73.5	(44.8–85.1)	57.0	(41.7–68.6)	54.8	(37.4–92.7)
BMI (kg/m^2^)	24.8	(17.4–40.0)	25.2	(19.3–32.1)	27.1	(20.6–34.8)	25.0	(19.2–33.4)	20.2	(16.4–22.4)	21.5	(14.7–30.7)
Waist Circumference (cm)	90.1	(65.7–125.1)	92.1	(79.4–113.1)	92.3	(83.7–119.9)	92.3	(76.9–112.1)	77.0	(63.8–90.8)	80.4	(62.6–107.8)
Glycemic factors												
FPG (mg/dL)	96	(75–242)	99	(81–168)	97	(87–155)	88	(82–102)	86	(79–96)	90	(69–147)
HbA1c (%)	5.8	(5.1–11.4)	5.7	(5.1–8.1)	5.6	(5.0–8.8)	5.4	(5.3–6.3)	5.4	(5.1–5.6)	5.6	(4.3–8.9)
HOMA–IR	1.6	(0.3–28.3)	1.3	(0.5–3.1)	1.1	(0.3–4.4)	1.2	(0.8–3.5)	1.0	(0.5–3.8)	1.0	(0.1–6.2)
Blood pressure												
Systolic BP (mmHg)	129	(94–190)	132	(114–186)	138	(108–178)	132	(116–152)	112	(92–121)	120	(82–178)
Diastolic BP (mmHg)	79	(56–127)	85	(66–129)	87	(71–116)	85	(66–104)	67	(55–73)	77	(46–115)
Lipid profile												
Triglycerides (mg/dL)	96	(26–394)	124	(43–663)	116	(57–673)	149	(42–232)	66	(39–121)	69	(20–462)
HDL–C (mg/dL)	58	(30–154)	59	(35–102)	57	(37–117)	61	(35–81)	71	(43–122)	66	(29–143)
LDL–C (mg/dL)	121	(34–249)	110	(50–157)	131	(56–169)	133	(92–172)	102	(49–150)	113	(49–207)
Liver-related parameters												
AST (U/L)	21	(11–99)	23	(14–76)	24	(14–131)	19	(12–36)	20	(13–59)	20	(11–162)
ALT (U/L)	22	(6–217)	24	(8–64)	32	(14–202)	18	(13–66)	13	(11–126)	16	(3–383)
γ–GTP (U/L)	25	(10–300)	45	(12–379)	78	(20–186)	21	(12–91)	21	(13–58)	20	(6–1019)
Platelet count (×10^4^/µL)	25.3	(15.0–54.6)	24.3	(12.7–49.8)	24.0	(14.7–41.2)	23.1	(18.5–31.4)	27.2	(18.1–32.8)	25.9	(11.5–52.3)
FIB-4 Index	1.00	(0.21–2.90)	1.12	(0.46–4.12)	0.96	(0.34–3.41)	0.98	(0.65–2.05)	0.70	(0.29–1.43)	0.93	(0.22–3.47)
M2BPGi (COI)	0.63	(0.21–2.03)	0.56	(0.16–1.29)	0.51	(0.25–1.78)	0.72	(0.32–1.30)	0.43	(0.18–1.66)	0.48	(0.13–2.93)
Elastography												
LSM (kPa)	4.6	(1.9–18.5)	4.5	(2.7–9.9)	4.8	(3.1–24.8)	4.6	(2.9–7.3)	4.4	(2.6–7.7)	4.1	(1.6–12.9)
LSM ≥ 7.0 (kPa), *n* (%)	31	(14.0%)	4	(9.8%)	2	(8.7%)	2	(18.2%)	1	(7.7%)	46	(7.2%)
CAP (dB/m)	286	(248–400)	286	(249–346)	289	(248–400)	279	(261–313)	270	(248–327)	197	(100–247)

Values are presented as median (range) or number (%). SLD, steatotic liver disease; MASLD, metabolic dysfunction-associated steatotic liver disease; MetALD, metabolic dysfunction-associated alcohol-related liver disease; ALD, alcohol-related liver disease; BMI, body mass index; FPG, fasting plasma glucose; HbA1c, hemoglobin A1c; HOMA-IR, homeostatic model assessment for insulin resistance; BP, blood pressure; HDL-C, high-density lipoprotein cholesterol; LDL-C, low-density lipoprotein cholesterol; AST, aspartate aminotransferase; ALT, alanine aminotransferase; γ-GTP, gamma-glutamyl transpeptidase; FIB-4, fibrosis-4 index; M2BPGi, Mac-2 binding protein glycosylation isomer; LSM, liver stiffness measurement; CAP, controlled attenuation parameter.

**Table 2:** Characteristics of parameters according to alcohol consumption in 10 g/day.

Alcohol Intake(g/day)	0 (*n* = 135)	0.1–10 (*n* = 72)	10.1–20 (*n* = 25)	20.1–30 (*n* = 15)	30.1–40 (*n* = 19)	40.1–50 (*n* = 9)	50.1–60 (*n* = 13)	60.1–70 (*n* = 9)	70.1–80 (*n* = 9)	80.1–90 (*n* = 4)
Lifestyle factors										
Caloric intake (kcal/day)	1751	1896	1967	2101	1990	2416	2519	2290	2362	2914
Anthropometric measures										
Body weight (kg)	64.9	68.5	62.7	68.7	71.3	71.9	74.6	74.2	73.1	68.4
BMI (kg/m^2^)	25.4	25.2	24.1	24.3	25.5	25.6	26.2	25.6	25.4	23.8
Glycemic factors										
HOMA–IR	2.0	2.4	1.8	1.5	1.6	1.1	1.7	1.4	1.7	0.8
Blood pressure										
Systolic BP (mmHg)	130	129	128	123	136	130	141	148	139	129
Diastolic BP (mmHg)	81	81	78	77	93	81	90	90	85	86
Lipid profile										
Triglycerides (mg/dL)	109	108	109	130	162	184	212	184	168	145
Liver-related parameters										
AST (U/L)	23	25	27	29	25	25	36	26	38	18
ALT (U/L)	25	33	30	30	27	26	41	31	49	20
γ–GTP (U/L)	29	39	46	59	53	56	98	75	116	47
M2BPGi (C.O.I)	0.74	0.60	0.60	0.57	0.58	0.71	0.60	0.56	0.69	0.45
Elastography										
CAP (dB/m)	293	290	282	278	291	280	301	300	292	279
Cardiometabolic risk factors										
Obesity, *n* (%)	119 (88.1%)	58 (80.6%)	19 (76.0%)	12 (80.0%)	15 (78.9%)	8 (88.9%)	12 (92.3%)	8 (88.9%)	8 (88.9%)	2 (50.0%)
Diabetes, *n* (%)	99 (73.3%)	49 (68.1%)	18 (72.0%)	9 (60.0%)	15 (78.9%)	4 (44.4%)	9 (69.2%)	5 (55.6%)	5 (55.6%)	3 (75.0%)
Hypertension, *n* (%)	85 (63.0%)	43 (59.7%)	14 (56.0%)	8 (53.3%)	17 (89.5%)	6 (66.7%)	11 (84.6%)	8 (88.9%)	8 (88.9%)	3 (75.0%)
Hypertriglyceridemia, *n* (%)	51 (37.8%)	22 (30.6%)	6 (24.0%)	7 (46.7%)	7 (36.8%)	5 (55.6%)	7 (53.8%)	5 (55.6%)	4 (44.4%)	3 (75.0%)
Low HDL–C, *n* (%)	47 (34.8%)	24 (33.3%)	5 (20.0%)	4 (26.7%)	3 (15.8%)	2 (22.2%)	1 (7.7%)	2 (22.2%)	2 (22.2%)	1 (25.0%)

Values are presented as mean values or number (%). BMI, body mass index; HOMA-IR, homeostatic model assessment for insulin resistance; BP, blood pressure; AST, aspartate aminotransferase; ALT, alanine aminotransferase; γ-GTP, gamma-glutamyl transpeptidase; M2BPGi, Mac-2 binding protein glycosylation isomer; CAP, controlled attenuation parameter.

**Table 3:** Correlation between clinical parameters and alcohol consumption.

	Correlation coefficient (r)	*p*-value
Demographics		
Age	−0.06	0.32
Lifestyle factors		
Caloric intake	0.39	<0.05
Sleep duration	0.04	0.45
Anthropometric measures		
Body weight	0.19	<0.05
BMI	0.00	0.97
Waist circumference	0.08	0.17
Glycemic factors		
FPG	0.02	0.72
HbA1c	−0.09	0.11
HOMA-IR	−0.12	0.03
Blood pressure		
Systolic BP	0.16	<0.05
Diastolic BP	0.21	<0.05
Lipid profile		
Triglycerides	0.28	<0.05
HDL-C	0.02	0.70
LDL-C	−0.08	0.14
Liver-related parameters		
AST	0.16	<0.05
ALT	0.09	0.10
γ-GTP	0.39	<0.05
Platelet count	−0.08	0.18
FIB-4 index	0.07	0.20
M2BPGi	−0.13	0.02
Elastography		
CAP	−0.01	0.91
LSM	0.03	0.55

Correlations were assessed using Pearson’s correlation coefficient. BMI, body mass index; FPG, fasting plasma glucose; HbA1c, hemoglobin A1c; HOMA-IR, homeostatic model assessment for insulin resistance; BP, blood pressure; HDL-C, high-density lipoprotein cholesterol; LDL-C, low-density lipoprotein cholesterol; AST, aspartate aminotransferase; ALT, alanine aminotransferase; γ-GTP, gamma-glutamyl transpeptidase; FIB-4, fibrosis-4 index; M2BPGi, Mac-2 binding protein glycosylation isomer; CAP, controlled attenuation parameter; LSM, liver stiffness measurement.

**Table 4:** Multivariable logistic regression analysis for fibrosis.

	OR	95% CI	*p*-Value	VIF
BMI	1.22	1.11–1.35	<0.01	1.31
HOMA-IR	1.15	0.99–1.35	0.08	1.13
HDL-C	1.00	0.98–1.02	0.83	1.16
Systolic BP	1.01	0.99–1.03	0.45	1.08

Collinearity was assessed using variance inflation factors (VIFs); all VIFs were ≤1.31, indicating no concerning multicollinearity. OR, odds ratio; CI, confidence interval; BMI, body mass index; HOMA-IR, homeostatic model assessment for insulin resistance; HDL-C, high-density lipoprotein cholesterol; BP, blood pressure.

## Data Availability

The data presented in this study are not publicly available due to privacy and ethical restrictions but are available from the corresponding author on reasonable request.

## Supplementary Materials

The following supporting information can be downloaded at: https://www.mdpi.com/article/10.3390/jcm15082860/s1. Table S1. Sensitivity analysis for fibrosis using an alternative threshold (LSM ≥ 6.0 kPa).

## Abbreviations

SLD, steatotic liver disease; MASLD, metabolic dysfunction-associated steatotic liver disease; MetALD, metabolic dysfunction-associated alcohol-related liver disease; ALD, alcohol-related liver disease; CAP, controlled attenuation parameter; LSM, liver stiffness measurement; BMI, body mass index; SBP, systolic blood pressure; DBP, diastolic blood pressure; TG, triglycerides; HOMA-IR, homeostatic model assessment for insulin resistance; SCFA, short-chain fatty acids; ASCVD, atherosclerotic cardiovascular disease; CVD, cardiovascular disease; MACE, major adverse cardiovascular events; BDHQ, brief-type self-administered diet history questionnaire; FDR, false discovery rate.
