# Supplementary material for: Alcohol Intake, Cardiometabolic Risk, Fibrosis, and Gut Microbiota in Steatotic Liver Disease: A Population-Based Health Checkup Study
Source: J Clin Med. 2026 Apr 9;15(8):2860. doi: 10.3390/jcm15082860 (PMC13116397; doi:10.3390/jcm15082860)
Supplement: Supplementary file 1 [file jcm-15-02860-s001.zip › jcm-4201180-supplementary.pdf]

Supplementary Table S1. Sensitivity analysis for fibrosis using an alternative threshold (LSM ≥ 6.0 kPa)

	OR	95% CI	p-value
BMI	1.21	1.11-1.32	<0.01
HOMA-IR	1.20	0.97-1.49	0.10
HDL-C	1.00	0.98-1.02	0.89
Systolic BP	1.00	0.99-1.02	0.70
